# Repeated heat pain threshold testing in pain-free individuals induces habituation and yields false-positive pre–post comparisons

**DOI:** 10.1038/s41598-026-61723-z

**Published:** 2026-07-16

**Authors:** David William Evans, Victoria Frowen, Michael Mansfield

**Affiliations:** School of Sport, Exercise and Rehabilitation Sciences, University of Birmingham, Edgbaston, Birmingham, B15 2TT UK

**Keywords:** Pain threshold, Heat: habituation, Adaptation, Asymptote, Health care, Medical research, Neuroscience, Psychology, Psychology

## Abstract

Heat pain thresholds (HPTs) are used for clinical assessments, sensory phenotyping, and pre–post comparisons. This usage assumes that HPTs are stable, exchangeable, and unaffected by repetition. Twenty consecutive HPT measures were taken at each volar forearm during three daily sessions in pain-free individuals. Negative exponential growth models were used to characterise HPT series and estimate effects of session, side, and time between sessions. To assess suitability for within-session pre–post comparisons, the mean of the first *n* repetitions of a series was compared with the mean of the subsequent *n* repetitions for *n* = 1–10, and ICC(3,1) calculated for *n* = 2–20. From 23 participants, 2266 HPT measurements were recorded within 118 series. Strong habituation was observed (Cohen’s *d* = 1.9), with HPT increasing by a mean of 4.9 °C across all series (95% CI: 4.4 to 5.3 °C, *p* <0.001). Repeated HPTs followed an asymptotic trajectory; lower initial HPTs were associated with larger repetition-related increases, and right forearms adapted faster than left. Later sessions were associated with higher initial and asymptotic HPT values and faster adaptation. Using single or averaged HPTs for within-series pre–post comparisons consistently yielded false-positive findings, while ICC decreased as *n* increased.

**Supplementary Information:**

The online version contains supplementary material available at 10.1038/s41598-026-61723-z.

## Introduction

Pain thresholds, the lowest intensity of a stimulus that induces pain^1^, are a widely used metric in pain research^2^. They are employed in the assessment of clinical conditions, such as fibromyalgia^3^ and pancreatic disorders^4^, and have been used to identify sensory phenotypes^5–7^. The quantitative sensory testing (QST) battery of standardised psychophysical tests^8^, designed to provide a comprehensive profile of somatosensory function, uses pain thresholds to assess sensory gain, specifically hyperalgesia and allodynia. Additionally, pain thresholds are used as pre–post ‘test stimuli’ within conditioned pain modulation (CPM) studies^9^, where increases in threshold following a ‘conditioning stimulus’ are interpreted as evidence of endogenous pain inhibition, with reduced CPM responses being typically interpreted as impaired inhibitory capacity.

Pain thresholds can be elicited via different stimulus modalities: thermal (heat or cold); mechanical (pressure or stretch); electrical; or chemical. Of these, heat pain thresholds (HPTs) are delivered through highly automated computer-controlled systems that minimise stimulus error. Heat stimuli are also perceived with short latency, since the cutaneous receptors that transduce heat stimuli encode and conduct signal rapidly^10^. These characteristics render HPTs well-suited for obtaining precise measurements.

HPTs are typically quantified as a single numerical value, derived either from a single measurement or by averaging multiple measurements taken at a single site^11–16^. Yet, averaging successive HPT measurements implicitly assumes that each is an exchangeable observation of the same underlying construct, independent of repetition order. This assumption has underpinned previous reports of HPT reliability^13,14,17^ being ‘good’^18^ or better. However, these estimates have been based on small repetition numbers.

The assumption that HPT is a stable metric has been challenged by the only previous study to have examined a larger number of successive HPT measurements^19^, which revealed progressively increasing values. In this context, increasing HPT values across successive repetitions represent a cumulative decrease in sensitivity: a phenomenon known as habituation^20,21^. This finding indicates that successive HPT measurements are not independent of repetition order and cannot be assumed to be exchangeable observations of a stable construct. Accordingly, increasing numbers of repetitions are likely to yield less interpretable reliability estimates. Moreover, pre–post HPT comparisons risk false-positive findings driven by repetition-induced changes rather than true experimental effects.

Importantly, HPT values appear to habituate with diminishing increments^19^, suggesting that they may level off at a limit (i.e., an asymptote)^21^. This would be biologically adaptive, presumably reflecting a mechanism that protects tissues from thermal damage. However, the number of repetitions studied so far (eight) has not been sufficient to demonstrate a clear asymptote. In addition, the duration of these adaptations remains uncertain. No adaptation remained when eight HPTs were repeated several days later (mean 5.2, SD: 3.2 days)^19^, suggesting spontaneous recovery^21^, whereas applying supra-threshold heat stimuli on consecutive days appears to create between-session habituation^22,23^. Accordingly, the longer-term trajectory and rate of recovery of HPTs remain unknown, as does any potential effect of order of testing at multiple sites, or laterality in which bilateral sites are tested. This study therefore aims to characterise the trajectory of a larger number of repeated HPT measurements in pain-free individuals, and the potential effect of conducting these repeated measurements at two symmetrical contralateral sites over multiple daily sessions.

## Methods

### Study aims

The primary aim of this study was to characterise how HPT changes over 20 repeated measurements, each separated by 30 seconds, at the same testing site. We hypothesised that repeated HPT measurement would be characterised by progressively diminishing increments, consistent with an asymptotic trajectory. Secondly, we aimed to investigate whether conducting repeated testing at two symmetrical contralateral sites (volar aspect of each forearm) in random order would reveal differences according to side and/or order of testing. We hypothesised that the second tested forearm would show evidence of habituation following prior testing of the first forearm, but that left and right sides would show no systematic difference. Thirdly, we aimed to assess whether habituation persisted across three sessions conducted on consecutive days. We hypothesised that HPT values would remain elevated across sessions, indicating between-session habituation. Fourthly, we aimed to evaluate the appropriateness of averaging successive HPT values at a single testing site. We hypothesised that means derived from earlier repetitions would differ systematically from means derived from the same number of later repetitions, indicating that successive repetitions should not be collapsed into a single undifferentiated average. Fifthly, we aimed to assess whether successive HPT repetitions could be treated as exchangeable observations of the same underlying construct, independent of repetition order, by examining reliability across increasing numbers of repetitions within a series. We hypothesised that successive repetitions would not be exchangeable, but would instead show systematic repetition-related change, which would in turn affect reliability over increasing numbers of repetitions.

### Study design

This observational laboratory study comprised an online onboarding survey followed by three laboratory HPT measurement sessions. Input for the design of the study was provided by a Patient and Public Involvement group of volunteers, particularly regarding the burden of testing on participants. The study was reviewed and approved by the Research Ethics Committee of the School of Sport, Exercise and Rehabilitation Sciences at the University of Birmingham (ref: MCR2425_03). Data were collected between February and March of 2025. All participants provided written informed consent via an electronic consent form.

### Recruitment

Convenience sampling was used to distribute the invitation, via emails and verbal invitations to colleagues, acquaintances, and friends. Social media posts and text invitations in group chats on various software platforms were also used. A snowball approach was utilised, where interested individuals were asked to pass on the study invitation to any of their contacts who may be willing to participate, even if they did not wish to participate themselves.

Interested individuals were provided with an open (publicly accessible and freely distributable) web link that directed respondents to an online survey, built using REDCap data collection software^24^ hosted on protected university servers. Individuals were first taken to a landing webpage containing all relevant information about the study, including aims, procedures, and potential risks and benefits. Using flow control logic, only once confirmation was provided that the study information was read and understood could individuals proceed to an online eligibility screening form. Similarly, only if self-declaring themselves as eligible could individuals progress to an online consent form.

Adults (≥18 years of age) were eligible to participate. Since an accurate measurement of pain thresholds requires an understanding of specific instructions^25^, individuals were ineligible if they declared themselves unable to understand spoken or written English. Participants rating their average current or recent (within the prior week) pain intensity at ≥20/100 on a numerical rating scale were also ineligible, as were individuals taking any medications that could affect their response to noxious stimuli (including analgesics, epilepsy drugs, or anti-depressants). We also excluded individuals who had a current or recent (within the last 3 months) serious injury (e.g., fracture), surgery, or pregnancy, and those who have had any previous injury, burn or surgery that caused scarring of the upper limbs (i.e., testing sites) or those with a current skin disorder (including eczema) that affected their upper limbs. Finally, we excluded anyone with a current central nervous system injury and/or impairment, and/or a current life-threatening or terminal illness with a short-life expectancy.

Once timestamped written informed consent was provided, participants were asked to complete a brief demographic questionnaire that included biological sex (assigned at birth), dominant upper limb (defined as that preferred for handwriting), ethnicity, educational attainment, smoking status, and current work status. Upon consenting, the participant was also sent an automated email to arrange a mutually convenient date/time for their laboratory sessions. Only participants who provided valid HPT data from at least one laboratory session were included in the analyses.

### Laboratory sessions

Each participant’s HPT testing took place at three laboratory sessions, which were intended to take place on consecutive days, at the same time of day. In case this 24-hour schedule was unattainable, the time (in hours) between sessions was measured and accounted for during statistical modelling. All HPT testing sessions took place within the same purpose-built university laboratory. The temperature of the laboratory was controlled and was recorded during each session using a digital room thermometer.

Participants were asked in advance to dress in clothing enabling access to both forearms and to remove any jewellery and watches. Participants were sat on a cushioned chair throughout each session. At each session, the testing procedure was explained in detail to the participant using a prepared script (see Supplement), and they were reminded of their right to withdraw at any time. Prior to commencement of testing, participants were asked to disclose the number of hours of sleep gained during the previous night, their caffeine intake (number of cups) since waking that day, and their current anxiety level (a single Likert scale item from 1 = ‘not at all anxious’ to 5 = ‘extremely anxious’).

Before testing commenced at the first session, participants were introduced to the testing apparatus, a Medoc TSA-II NeuroSensory Analyzer thermal stimulation device (Medoc Ltd, Israel), which is CE-certified, widely used, and capable of safe, rapid (temperature increase/decrease rate of up to 8 °C/s) topical heat stimuli with a built-in safety heat limit of 50.5 °C to avoid thermal injury even when measurements are repeated^26,27^. For all HPT tests, a metal 30x30 mm Peltier thermode was attached to the skin of the testing site using an elastic Velcro strap, which was reinforced by loosely wrapped self-adhering medical bandage to avoid any thermode movement whilst ensuring there was no significant pressure stimulus. All HPTs were measured using an ascending limits approach^28^. The heat stimulus gradually increased at a rate of 1 °C/s, from a baseline temperature of 32 °C, towards the maximum attainable temperature of 50.5 °C.

During the first session only, participants were familiarised with HPT measurement via a single test on the anterior shin on the same side as the previously defined dominant upper limb. If participants did not provide an upper limb dominance, the right shin was used for familiarisation testing. For forearm testing, two pillows were placed upon the participant’s lap to provide comfortable support for their upper limbs. They were asked to keep their elbows relaxed in a flexed position (approximately 90°) with their wrists relaxed and gently supinated. At the first session, using a cloth tape measure, the midpoint between the bicep tendon insertion and the proximal wrist crease was measured in both upper limbs. At each midpoint, a 30x30 mm square was drawn onto the skin of the volar forearm using a surgical skin-marking pen (Schuco Ltd, United Kingdom) and a pre-cut plastic stencil. Each of these 30x30 mm drawn squares indicated a testing site that would match the footprint of the thermode. The drawn square was expected to be visible for 2-3 days but was re-drawn at the second session if seen to be fading, ensuring that precisely the same areas of skin were tested at all sessions.

At every session, HPT measurements were repeated up to 20 times at each forearm testing site, marked by the drawn squares. This constituted a single series. The thermode remained attached to the skin at each testing site until all HPT repetitions of the series were completed at that site during that session. At each forearm site, repetitions were separated by a 30-second inter-stimulus interval to reduce the likelihood of temporal sensitization via ‘wind-up’^29,30^. Participants were instructed to close their eyes during each HPT test to help retain their focus on the heat stimuli. During inter-stimulus intervals, participants were permitted to open their eyes but were asked to close them again approximately 5-10 seconds before the thermode temperature began to increase for the next test.

During forearm testing, participants held a response button in the hand contralateral to the forearm being tested. Scripted instructions were read out prior to commencement of testing at each site, specifying that the button should be pressed “*at the moment the heat becomes painful*” (see Supplement for full script). As soon as the participant pressed the response button, the temperature commenced its descent towards the baseline temperature at a rate of 8 °C/s. If the ascending heat had not induced pain once the safety limit of 50.5 °C was reached, the temperature would automatically retreat to the baseline of 32 °C and no HPT value would be recorded for that repetition. In this situation, the series would be truncated and no further repetitions would be conducted at that testing site during that session.

The same process was then repeated on the testing site at the second forearm. The order in which forearm sites were tested was randomised within REDCap and this order was automatically recorded. A minimum interval of 5 minutes was used between testing sites to allow equipment to be transferred from one forearm to the other. All laboratory session data were manually entered into REDCap (one HPT value at a time, as each was gained) and data were uploaded to the server after testing was completed at each site to minimise the chance of data loss. Any errors or adverse events were noted.

### Sample size

Sample size calculations were performed using healthy participant data from Santos Agostinho et al^19^, which indicated an approximate mean HPT increase of 3.0 °C (SD: 3.5 °C) between the first and sixth repetition, corresponding to a Cohen’s *d* of 0.86. This calculation suggested that a minimum of 19 participants would be required to achieve 90% power at α = 0.05 in a within-subjects repeated measures design. To confirm the adequacy of this estimate, we fitted mixed-effects models with random intercepts and slopes to the individual participant data^19^. Parameter estimates from these models were used to generate simulated datasets reflecting our planned study design (20 HPT repetitions per side, three sessions per participant). Power was then assessed across a range of sample sizes using the ‘simr’ package in R, with 1000 simulations performed for each sample size condition. The mean observed power for detecting the effect of repetition exceeded 99% even with 10 participants, providing reassurance for our sample size estimates. To ensure robust statistical power across a range of potential data conditions, we retained our original target of at least 19 participants for the primary analysis.

### Statistical analysis

Participants, sessions, series and repetition numbers were summarised. To quantify within-series HPT change, Cohen’s *d* for paired data was calculated as the mean difference between first and last repetitions of a series, divided by the standard deviation (SD) of all first-to-last paired differences. The magnitude of Cohen’s *d* was interpreted using Sawilowsky’s categories^31^: very small (0.10 ≤ *d* < 0.20), small (0.20 ≤ *d* < 0.50), medium (0.50 ≤ *d* < 0.80), large (0.80 ≤ *d* < 1.20), very large (1.20 ≤ *d* < 2.00), and huge (*d* ≥ 2.00).

Because progressively smaller increases in HPT values have previously been observed across repeated measurements^19^, HPT was modelled using asymptotic non-linear models. The asymptotic function was specified as a negative exponential growth curve^20,21^:

1 $$HP{T}_{ij}= L - \left(L - I\right){e}^{-kr}$$

where $HP{T}_{ij}$ is the predicted heat pain threshold for measurement *i* in participant *j*; *L* represents the asymptotic HPT or limit; *I* is the initial HPT at the first repetition; *k* is the rate constant governing how rapidly HPT approaches its limit *L* across successive repetitions; and *r* is the repetition number, re-indexed so that the first repetition number corresponds to 0.

First, a pooled negative exponential growth model was fitted using the nls() function in base R, treating all observations as arising from a single overall trajectory without participant-level random effects. This initial model was used to assess the suitability of the asymptotic functional form within the negative exponential growth model (Equation 1) and to obtain initial values of *L*, *I* and *k* to carry forward to subsequent models. Non-linear mixed-effects models were then fitted at participant level, using the nlme() function from the ‘nlme’ R package^32^, with HPT modelled as a continuous outcome variable and repetition number (0–19) incorporated directly into the negative exponential function through the re-indexed repetition term *r*. Sparse models were first fitted with participant-level random effects on *L* and *I*, and then on *L*, *I*, and *k* without covariates. A fuller candidate model was then fitted, in which session number (1–3) was modelled as a determinant of *L* and *I*, and forearm side (right, left), order of forearm testing within each session (first, second), time since the first session (hours), and biological sex (male, female) were modelled as determinants of *I*, while *k* was initially retained as an intercept-only fixed effect. Subsequent models tested whether selected covariates further improved fit when modelled as determinants of *k*, including session number, biological sex, and forearm side. Models were fitted using maximum-likelihood estimation. Model fit was compared using Bayesian Information Criterion (BIC), which favours more parsimonious models, and the best-supported model was carried forward. Fixed-effect estimates were reported with associated standard errors, *t*-values and *p*-values. Model assumptions, including adequacy of the negative exponential functional form and normality of residuals, random effects, and homoscedasticity of residuals, were assessed using diagnostic plots. Correlations were interpreted using the following categories^33^: negligible: 0 ≤ |*r*| < 0.10; weak: 0.10 ≤ |*r*| < 0.40; moderate: 0.40 ≤ |*r*| < 0.70; strong: 0.70 ≤ |*r*| < 0.90; very strong: 0.90 ≤ |*r*| ≤ 1.

If the Medoc TSA-II device’s safety limit of 50.5 °C was reached and no HPT value was recorded for a given repetition, with any remaining repetitions in the series consequently unattempted, the primary analysis would treat both the limit-reaching observation and the subsequent unattempted repetitions as missing because of protocol-imposed termination. In such an event, a sensitivity analysis would be performed, in which limit-reaching non-response observations would be treated as right-censored at 50.5 °C, while the subsequent unattempted repetitions would remain missing to examine whether the main conclusions from the primary analysis were materially affected by this data handling choice.

To replicate and evaluate the current practice of averaging successive HPT values^11–16^, the mean HPT of the first *n* repetitions was compared with the mean of the subsequent *n* repetitions for all *n* ranging from 1 to 10. In other words, we compared the mean of repetitions 1 to *n* with the mean of repetitions *n*+1 to 2*n*, for all values of *n* between 1 and 10. For each *n*, paired *t*-tests were conducted within each session and side, comparing the means for each participant. We did not adjust for multiple comparisons, since the purpose was to emulate the findings of separate independent studies using different values of *n* to illustrate how false-positive findings may arise when successive HPT values are averaged. Effect sizes (Cohen’s *d*), *t*-statistics and *p*-values were calculated for all comparisons. Consistent significant differences (*p* < 0.05) yielded from these comparisons would indicate that averaging consecutive repetitions yields false-positive findings.

Intra-class correlation coefficients (ICCs) were also calculated, using the first *n* repetitions within each series from *n* = 2 to 20, to assess the within-session reliability of repeated HPT measurements. Using the ‘irr’ R package^34^, a two-way mixed-effects model, ICC(3,1) under Shrout and Fleiss’s convention^35^, was used to estimate the absolute agreement of measurements for each side and session. Declining ICC values across increasing repetition count *n* would indicate that the proportion of variance attributable to between-subject differences decreases relative to within-series variability; this would suggest that successive repetitions are not identically distributed measurements of a single stable construct, making ICC difficult to interpret as a reliability estimate.

## Results

Thirty-six individuals responded to the study invitation, of which 23 consented and participated in one or more laboratory sessions (Table 1). Of the 23 participants, 14 (60.9%) were female and 9 (39.1%) were male. The mean age of participants was 23.7 (SD: 2.1) years. On average, participants attended 2.6 (SD: 0.6) sessions: 14 participants (60.9%) attended all three sessions; 8 participants (34.8%) attended only two sessions; and 1 participant (4.3%) attended only one session. Not all participants were able to attend sessions at 24-hour intervals; the mean time from the commencement of the first to the second session was 47.5 (SD: 27.3) hours, and from first to third session was 69.6 (SD: 36.3) hours. Each session took approximately 1 hour to complete, and the mean room temperature was comfortable (22.4 °C) and consistent (SD: 0.89 °C).

**Table 1:** Demographics and session characteristics of participants.

**Characteristic**	**Level**	**Value**
Years of age, mean (SD)	All	23.7 (2.1)
Sex, n (%)	Female	14 (60.9)
	Male	9 (39.1)
	Prefer not to say	0 (0)
Dominant upper limb, n (%)	Right	22 (95.7)
	Left	1 (4.3)
	Both	0 (0)
Ethnicity, n (%)	White	13 (56.5)
	Asian or Asian British	5 (21.7)
	Chinese or Chinese British	1 (4.3)
	Black or Black British	0 (0)
	Other ethnic group	4 (17.4)
	Prefer not to say	0 (0)
Age left full-time education, n (%)	Still in full-time education	21 (91.3)
	>20 years	2 (8.7)
	17-20 years	0 (0)
	16 years	0 (0)
	Prefer not to say	0 (0)
Employment, n (%)	Not employed	15 (65.2)
	Part time	7 (30.4)
	Full time	1 (4.3)
History of smoking, n (%)	Never smoked	21 (91.3)
	Ex-smoker	2 (8.7)
	Current smoker	0 (0)
Sessions attended, n (%)	3 sessions	14 (60.9)
	2 sessions only	8 (34.8)
	1 session only	1 (4.3)
Laboratory temperature, mean (SD)	Session 1	22.4 (0.9)
	Session 2	22.3 (0.9)
	Session 3	22.3 (1.0)
Sleep (hours) night before session, mean (SD)	Session 1	7.2 (1.0)
	Session 2	7.1 (1.5)
	Session 3	7.9 (0.9)
Caffeine (cups) on day of testing, mean (SD)	Session 1	0.8 (0.7)
	Session 2	1.0 (0.7)
	Session 3	1.0 (0.9)
Anxiety (single item), mean (SD)	Session 1	1.6 (0.8)
	Session 2	1.4 (0.7)
	Session 3	1.9 (1.2)

From the 23 participants, a total of 2,266 HPT measurements (from a potential 2,760) were recorded, spanning 118 series of repetitions across all sessions. Of these, 110 series (93.2%) were fully complete, comprising all 20 planned repetitions, with participants completing a mean of 4.8 series each (SD: 1.3). Five participants reached the safety limit of 50.5 °C without reporting a painful response, resulting in 6 limit-reaching non-response observations; these series were then truncated, and a further 77 measurements were therefore not attempted. For one participant, during their third session, the series on the second tested (right) forearm was aborted after 5 repetitions because of equipment malfunction and was not re-attempted. For another participant, the 20^th^ HPT value was inadvertently not entered for the left (first tested) forearm at their first session. No adverse events occurred during or after testing, including no lasting discomfort or visible skin injury.

**Fig. 1:**
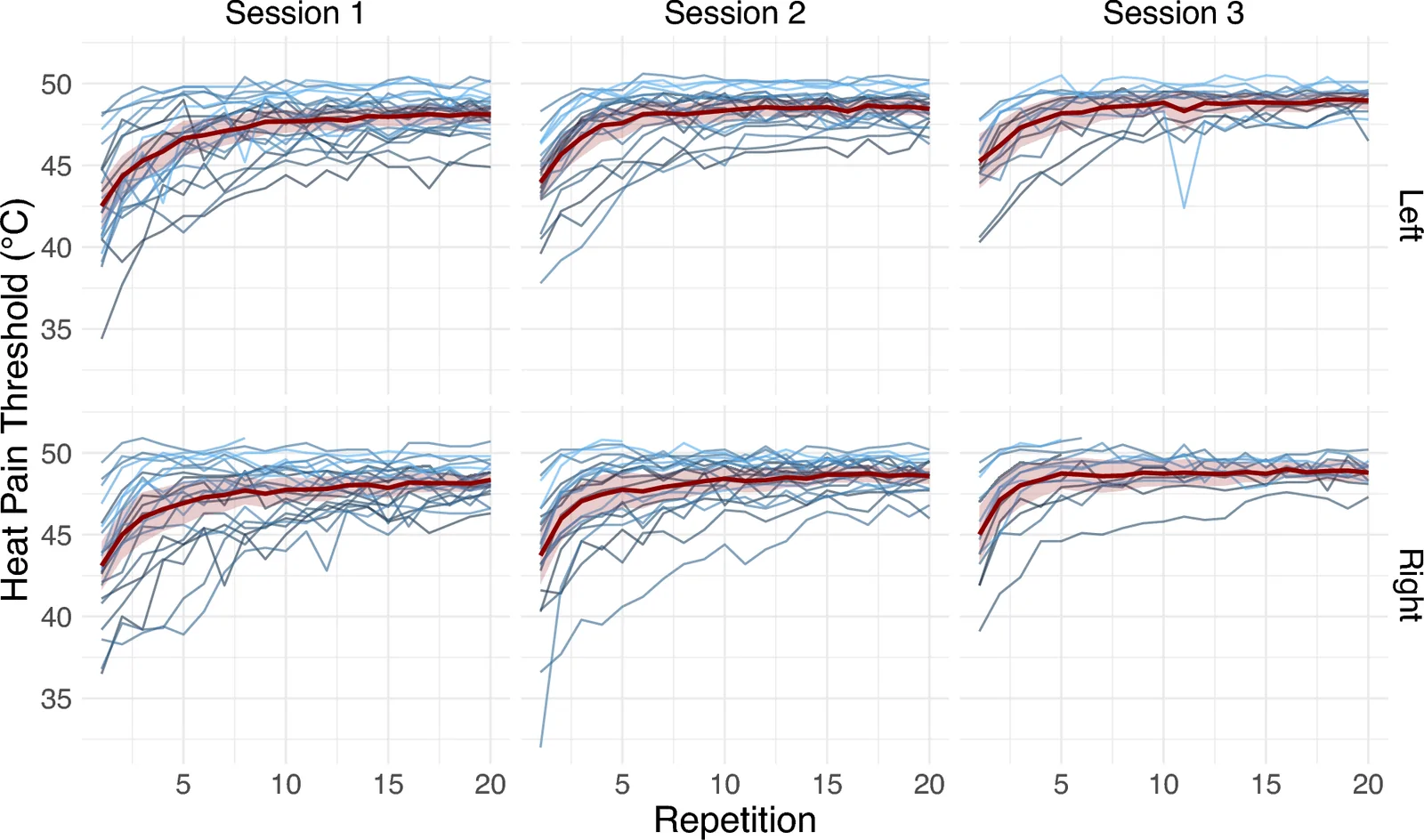
Mean (95% CI) and individual HPT values (°C) across repetitions, sessions and sides.

The overall mean HPT value, across all repetitions, sides and sessions was 47.68 °C (SD: 2.24 °C). The mean difference between first and last recorded HPT values across all series of repetitions was 4.85 °C (95% CI: 4.38 to 5.32 °C, *p* <0.001), which corresponds to a ‘very large’ within-series habituation effect (Cohen’s *d* = 1.87)^31^. Figure 1 displays mean HPT values with 95% CIs for each repetition at each site within each session, showing a non-linear within-series increase in mean HPT over successive repetitions, with the curves flattening in a manner consistent with an asymptotic trajectory.

The initial pooled asymptotic model supported the suitability of a negative exponential growth trajectory for repeated HPT values by converging successfully and yielding plausible estimates for the initial HPT (*I*), asymptotic HPT (*L*), and rate constant (*k*). Allowing participant-level random effects on *L*, *I*, and *k*, compared with random effects on *L* and *I* only, improved model fit (BIC = 7296.3 vs 7411.3). Adding fixed effects for session, time since first session, forearm side, order of testing, and biological sex then markedly improved fit (BIC = 6841.3). Removing order of testing from *I* slightly improved fit by producing a more parsimonious model (BIC = 6835.7). Allowing session to influence *k* improved model fit further (BIC = 6767.8). However, adding biological sex to both *I* and *k*, or to *k* alone, did not improve fit (BIC = 6774.8 and 6768.1, respectively); thus, sex was not retained in the final model. Allowing forearm side to influence *k* improved fit further still (BIC = 6755.8), and removing side as a determinant of *I* improved model fit again (BIC = 6750.3). Hence, the retained model was a negative exponential mixed-effects model with participant-level random effects on *L*, *I*, and *k*, with session modelled as a determinant of *L*, *I*, and *k*, time since first session as a determinant of *I*, and forearm side as a determinant of *k*.

HPT values also increased between sessions, indicating between-session habituation. In session 1, the estimated initial HPT was 42.87 °C (SE = 0.55 °C). Relative to session 1, the estimated initial HPT (*I*) was 3.16 °C higher in session 2 (SE = 0.27 °C, *p* < 0.001) and 4.78 °C higher in session 3 (SE = 0.39 °C, *p* < 0.001). Alongside these session effects, there was a statistically significant reduction of 0.041 °C in *I* for each additional hour since the commencement of session 1 (SE = 0.005 °C, *p* < 0.001), equivalent to 0.98 °C per day. The estimated asymptotic HPT (*L*) in session 1 was 48.38 °C (SE = 0.20). Relative to session 1, *L* was 0.25 °C higher in session 2 (SE = 0.06 °C, *p* < 0.001) and 0.43 °C higher in session 3 (SE = 0.08, *p* < 0.001). The rate constant *k* was 0.417 in session 1 (SE = 0.058, *p* < 0.001). Relative to session 1, *k* was 0.076 greater in session 2 (SE = 0.013, *p* < 0.001) and 0.173 greater in session 3 (SE = 0.029, *p* < 0.001), indicating a progressively faster approach to the asymptote across sessions. Right forearms also demonstrated a greater *k* than left forearms (0.045, SE = 0.007, *p* < 0.001), indicating more rapid habituation on the right side.

Diagnostic plots supported the adequacy of the negative exponential functional form and showed acceptable residual normality, random-effect normality, and residual homoscedasticity, with no major structural departures from model assumptions. In the sensitivity analysis, treating the 6 safety-limit reaching non-response HPT observations as 50.5 °C while leaving subsequent unattempted repetitions as missing, the parameter estimates from the final model were virtually unchanged, indicating that the results were robust.

**Fig. 2:**
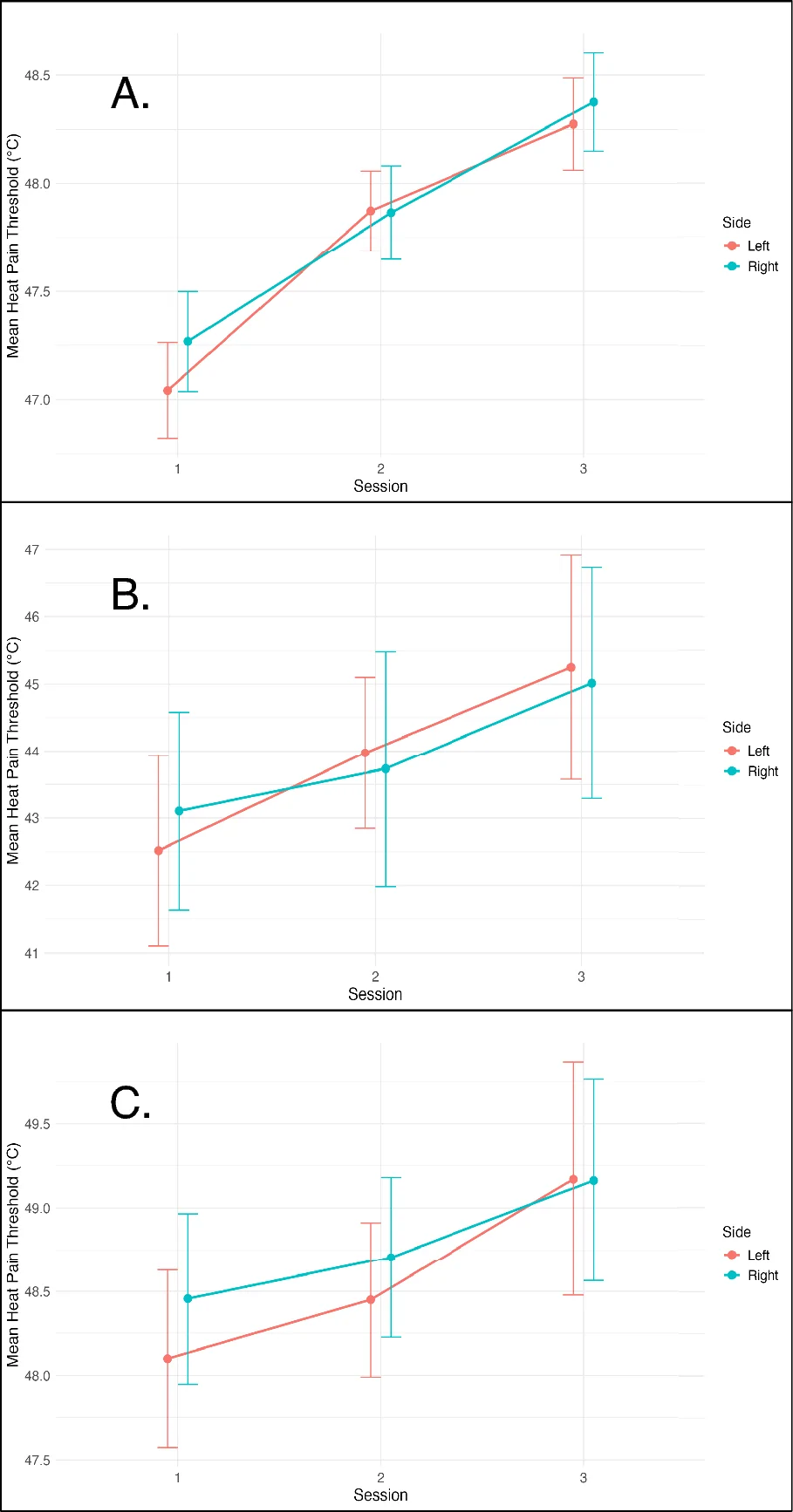
Mean HPT values (°C) within sessions (**A**) mean of all HPT repetitions within each session; (**B**) mean of first HPT measurement within each session; (**C**) mean of last HPT measurement within each session.

Figure 2 presents mean values of the first repetition, last repetition (20 when participants provided complete data), and all repetitions of each series, illustrating the progressive and consistent increase in HPT between sessions.

**Fig. 3:**
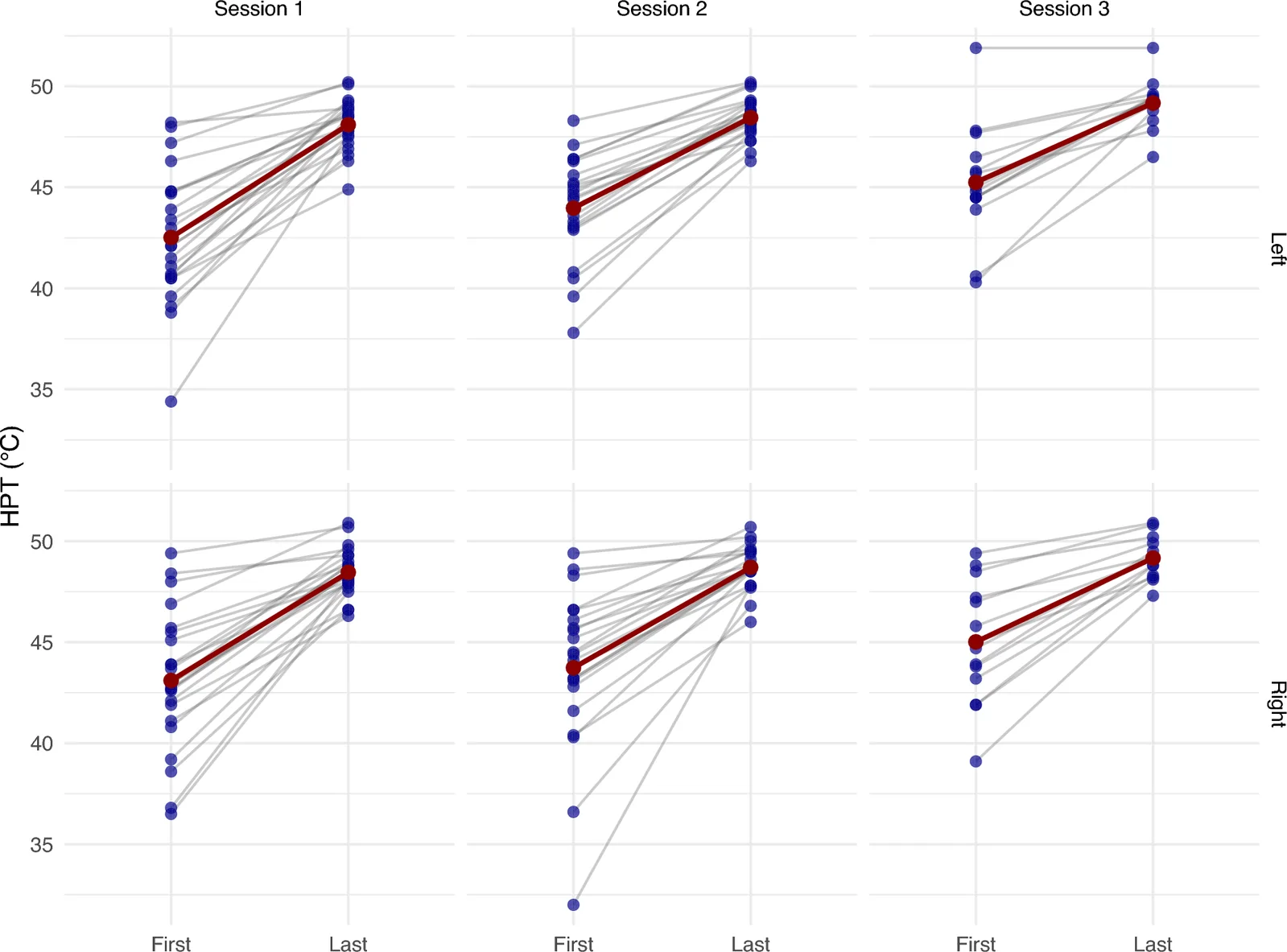
Paired first and last HPT values.

Within the final model, between-participant variability was greater for *I* (SD = 2.57 °C) than for *L* (SD = 0.94 °C), indicating that participants differed more in their initial HPT values than in their asymptotic limits. *I* and *L* were strongly positively correlated (*r* = 0.771) ^33^. In addition, *I* was very strongly negatively correlated with the *L*–*I* difference (estimated *r* = -0.95), indicating that participants with lower initial HPT values increased more than those with higher initial values. Figure 3 displays paired first and last HPT values per series for each session and side, confirming that participants with lower initial HPT values reached final values close to those of participants starting with higher initial values.

**Fig. 4:**
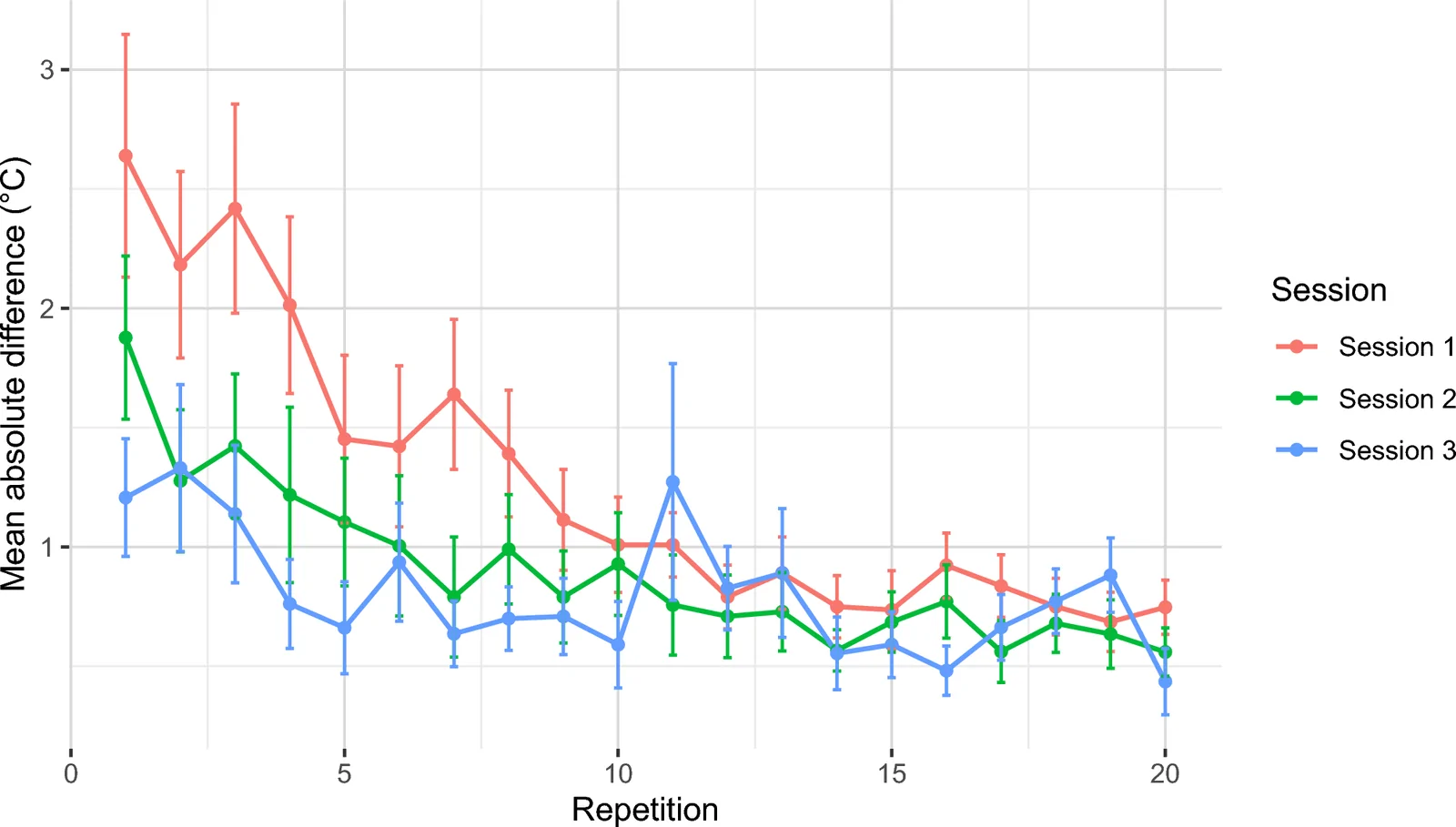
Differences between paired left and right forearm HPT values.

The side difference identified in the mixed-effects model was explored further. Figure 4 shows that left–right differences were greatest in the earliest HPT repetitions within each session, diminishing with subsequent repetitions and stabilising after approximately 10 repetitions. These left–right HPT differences also reduced over successive sessions, with less pronounced early-repetition differences in sessions 2 and 3 than in session 1.

**Fig. 5:**
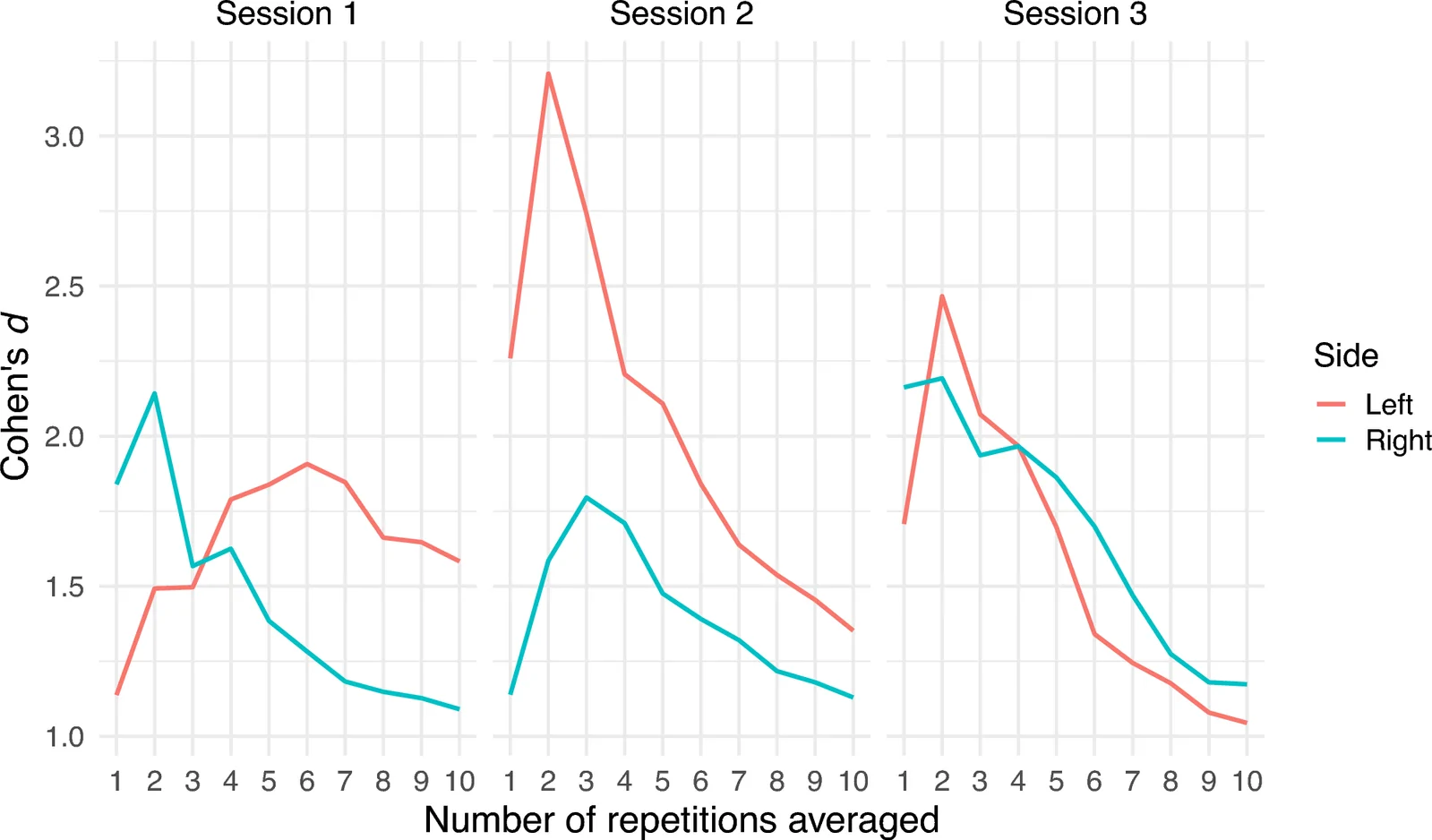
Effect size (Cohen’s *d*) as a function of the number of averaged HPT repetitions.

A detailed comparison of mean values for different sizes of repetition counts (*n* = 1 to 10) of HPT measurements, from the paired *t*-tests, is presented in the Supplement. In summary, for every repetition count (*n*) tested, at both sites and at every session, averaging successive HPT values produced a statistically significant difference (all paired *t*-tests, *p* < 0.01). The mean HPT of the first *n* repetitions was significantly lower than that of the next *n* repetitions for all tested values of *n*. Figure 5 displays the effect size (Cohen’s *d*) for each value of *n* from these comparisons. Notably, the effect size generally peaked between *n* = 2 and *n* = 3 repetitions, before diminishing steadily as *n* increased further.

**Fig. 6:**
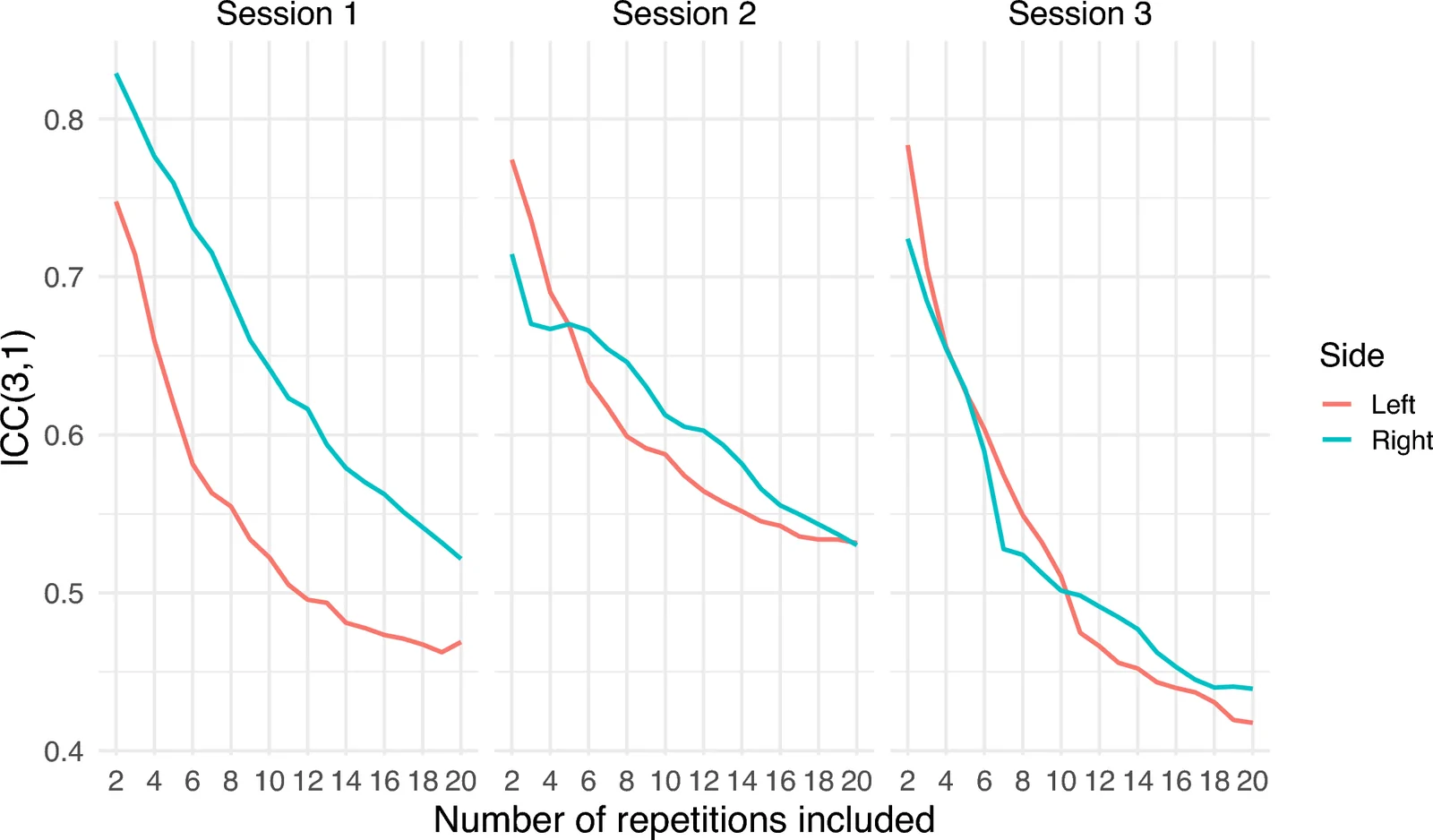
Intra-class correlation as a function of the number of HPT repetitions.

Similarly, Figure 6 displays the intra-class correlation (ICC^1,3^) for each number of repetitions from *n* = 2 to 20. ICC values can be seen to decline systematically with increasing *n*. At *n* = 2, all ICC(3,1) values exceeded 0.70, indicative of ‘good’ to ‘excellent’ reliability^18^, but as *n* increased towards 20 they fell below 0.55, indicating only ‘fair’ reliability.

## Discussion

This study extends previous important findings that successive HPT values habituate when repeated measurements are taken at the same site^19^. Fulfilling our primary aim, and in line with our first hypothesis, our results indicate that repeating HPT measurements produces progressively smaller increments until values approach a plateau or asymptote, which are features characteristic of habituation^21^. We propose the term ‘HPT_limit_’ (represented by *L* in Equation 1) to describe the value that an individual’s HPT would approach if repetitions were extended indefinitely, which likely reflects underlying biological factors. By contrast, we propose the term ‘HPT_max_’ to refer to the highest observed HPT value within a given series, serving as an empirical approximation to HPT_limit_ whilst inevitably being constrained by the testing protocol employed, such as the number of repetitions applied and the maximum available heat stimulus temperature. Although the biological significance of HPT_limit_ is not yet fully understood, it likely represents a physiological or perceptual upper bound for HPT. For an intact nervous system, it would be safe to assume that HPT cannot increase indefinitely^21^ to ensure that pain is appropriately experienced at temperatures known to damage the skin^26^.

It would be reasonable to expect that individuals with lower ‘HPT_initial_‘ (represented by *I* in Equation 1) values would also plateau at a lower temperature (i.e., exhibit a lower HPT_limit_), implying that participants would habituate to a similar extent. However, this was not observed. Instead, individuals with lower HPT_initial_ values demonstrated larger repetition-related increases to reach similar HPT_limit_ values to those with higher HPT_initial_ values. This finding challenges current conventional wisdom in two important ways. Firstly, the current definition of pain^36^ describes it as being “associated with, or resembling that associated with, actual or potential tissue damage.” From this definition, lower HPT_initial_ values could reasonably be interpreted as indicating susceptibility to tissue damage at a lower temperature; intuitively, one might therefore expect these individuals to exhibit less pronounced habituation to offer greater protection to their tissues. Yet, our results suggest the opposite: greater habituation in those with lower HPT_initial_ values. Secondly, lower HPT_initial_ values have traditionally been interpreted as indicating heightened pain ‘sensitivity’^8,37^, implying a more protective and potentially maladaptive pain response^38,39^. Our results, showing that individuals with lower HPT_initial_ exhibit greater habituation, perhaps reflects a more flexible neurophysiological response profile through stronger engagement of spinal cord modulation, descending inhibition, or attentional modulation. Conversely, higher HPT_initial_ values, associated with smaller increases in HPT with repeated stimulation, perhaps reflect a more static response profile.

Contrary to reference values based on averaged HPTs, which consistently report lower values in females^8,40^, biological sex did not improve model fit when tested as a determinant of HPT_initial_ and/or the rate constant *k*, and was therefore not retained in our final model. Contrary to our earlier hypothesis, we observed significant left–right differences in the rate at which HPT_limit_ was approached, with right forearms demonstrating more rapid habituation than left. All but one participant was right-handed; hence, in line with previous stimulus-response studies^41^, limb dominance could well be the primary explanation here. The underlying mechanism could be peripheral, with dominant upper limbs receiving more frequent stimulation during activities of daily living^42,43^. Equally, limb dominance arises from hemispheric lateralisation of motor control systems within the cerebral cortex^44^, suggesting involvement of central mechanisms. Irrespective, these differences diminished with repeated stimulation, leading to greater similarity between sides for later repetition values. This indicates that different body areas of an individual, even when symmetrical, cannot be assumed to yield comparable HPT_initial_ values or adaptation trajectories, although HPT_limit_ values appear to be more consistent across sides. This finding is particularly important for future studies that desire pre–post pain threshold testing: bilateral mirror-image testing sites do not yield comparable baseline values and therefore cannot be used as a substitute to same-site testing.

Peripheral nociceptor fatigue may explain within-session habituation following repetitive noxious heat stimuli^19^. A *δ*-fibre firing rates have been shown to diminish with repeated stimulation, consistent with receptor fatigue, whereas unmyelinated C-fibres appear more resistant to such fatigue^45^. However, we deliberately employed a 30-second inter-stimulus interval to avoid ‘wind-up’, which is primarily due to the prolonged excitatory postsynaptic potentials evoked by C-fibre stimulation^29,30,46^. Moreover, if peripheral fatigue were to entirely account for the observed habituation, complete receptor recovery between sessions would be expected, resulting in no session-to-session difference in HPT values. Instead, the association of session with HPT_initial_, HPT_limit_, and the rate constant *k* is more consistent with a centrally mediated learning effect^22,23,47–49^, while the association of forearm side with *k* suggests that lateralised factors may also influence the dynamics of adaptation. Independent of wind-up^50^, high-intensity heat stimuli can induce facilitated responses in dorsal horn neurons via central sensitization^51,52^, which may compete with the observed within- and between-session habituation of HPT. That said, we also found a significant time-dependent reduction in HPT_initial_, of 0.984 °C per day between sessions, a partial offset rather than a reversal or absence of between-session habituation, suggesting a gradual regression of the stimulus-induced adaptation over time consistent with spontaneous recovery of habituation^21^. Together, these findings support the view that HPT_initial_, HPT_limit_, and *k* capture related but distinct biological aspects of heat pain processing.

Our findings clearly demonstrate that HPT measurements are dynamic, and that – contrary to convention – they cannot be adequately represented by a single summary value. We have shown that comparing the mean of any number of successive HPT values to that of an equal number of subsequent repetitions consistently yields statistically significant differences purely as an artefact of repeated pain threshold testing. Notably, the average effect size associated with these false-positive within-session results peaked at 2–3 repetitions, which is the number commonly averaged in pre–post comparisons. A clear implication of this finding is that within-session HPT testing cannot be trusted to represent a true effect of an intervention or conditioning stimulus, such as those used within CPM paradigms^9^, in which test stimuli must be stable and unaffected by the influence of prior or subsequent measurements. Similarly, we observed steadily decreasing ICC(3,1) values as successive HPT repetitions were added to the analysis. This pattern is consistent with systematic non-stationarity across repetitions, whereby successive HPT repetitions do not represent exchangeable observations of a single stable underlying construct. Accordingly, the resulting ICC values are difficult to interpret as reliability estimates. Previously reported ‘good’ reliability of repeated HPTs^13,14^ is likely to reflect the limited influence of habituation when only very short repetition series were used, rather than an inherent stability of the measure itself.

These results have important implications for how HPT measurements are recorded, analysed, interpreted, and reported. Future studies should define the specific construct of interest explicitly, whether this is HPT_initial_, HPT_limit_, the rate of adaptation *k*, or another derived parameter. Researchers should avoid averaging across successive repetitions that are expected to change systematically, unless the selected repetition or repetition block corresponds to a prespecified construct. Reliability analyses should then be matched to that construct, rather than applied naively to raw successive repetitions. Where sufficient HPT repetition numbers are feasible, full HPT trajectories should be modelled directly and parameters such as HPT_initial_, HPT_limit_, and *k* should be reported. Where only a small number of repetitions is feasible, investigators should prespecify exactly which repetition or repetition block constitutes the outcome and interpret this as a protocol-specific estimate, rather than as an exchangeable average of stable repeated measurements. Reliability analyses should then be matched to the specified construct, rather than applied naively to raw successive repetitions.

Far from diminishing the value of HPT testing, our findings extend its potential utility. Most previous studies have estimated only HPT_initial_ values, and extrapolated inferences from these. Yet, HPT_limit_ and the rate of adaptation *k* – previously overlooked parameters revealed only through repetitive testing – may have significant biological importance and could be crucial for understanding underlying mechanisms of adaptation and phenotyping individuals. Given that our findings are based on healthy, young volunteers, these dynamic parameters of HPT should next be investigated in clinical populations, where conditions such as fibromyalgia may exhibit different adaptive responses to those we have observed. Additionally, this adaptive behaviour has so far only been observed with heat stimuli; equivalent studies using other stimulus modalities are needed to assess whether similar adaptations occur with other modalities.

It is worth noting that our study incorporated an equipment safety limit of 50.5 °C, which was surpassed without pain by five participants during testing. Consequently, the mean value of HPT_max_ might have been higher had this safety constraint not been in place. Because HPT_limit_ was estimated from observations obtained under this constraint, it should likewise be interpreted in the context of this upper temperature limit. It is also important to point out that the HPT values reported in our study may be specific to the testing conditions used, such as the ascending heating rate (1 °C /s) and inter-stimulus interval (30 seconds)^21^. Future studies should systematically vary such conditions to assess whether adaptations vary as a result.

## Conclusion

HPTs strongly habituate when repeatedly measured in pain-free individuals. HPT habituation can occur both within and between testing sessions. Individuals with lower initial HPT values showed larger repetition-related increases than those with higher initial values to approach similar asymptotic values. Using single or averaged HPT values for within-session pre–post comparisons consistently yields false-positive results and should be avoided. Because successive HPT repetitions cannot be assumed to provide exchangeable measurements of a stable construct independent of repetition order, ICC applied naively to raw HPT values is not readily interpretable as a reliability metric. Future studies should define explicitly whether the construct of interest is the initial HPT, the asymptotic limit, the rate of adaptation, or another derived parameter.

## Supplementary Information

Supplementary Information 1.

Supplementary Information 2.

## Data Availability

Data are provided in the supplementary information files.
