# Supplementary material for: Repeated heat pain threshold testing in pain-free individuals induces habituation and yields false-positive pre–post comparisons
Source: Sci Rep. 2026 Jul 16;16:26806. doi: 10.1038/s41598-026-61723-z (PMC13518313; doi:10.1038/s41598-026-61723-z)
Supplement: Supplementary file 1 [file 41598_2026_61723_MOESM2_ESM.pdf]

Supplementary material

Figure S1. Mean (95% CI) and individual HPT values (°C) of males and females

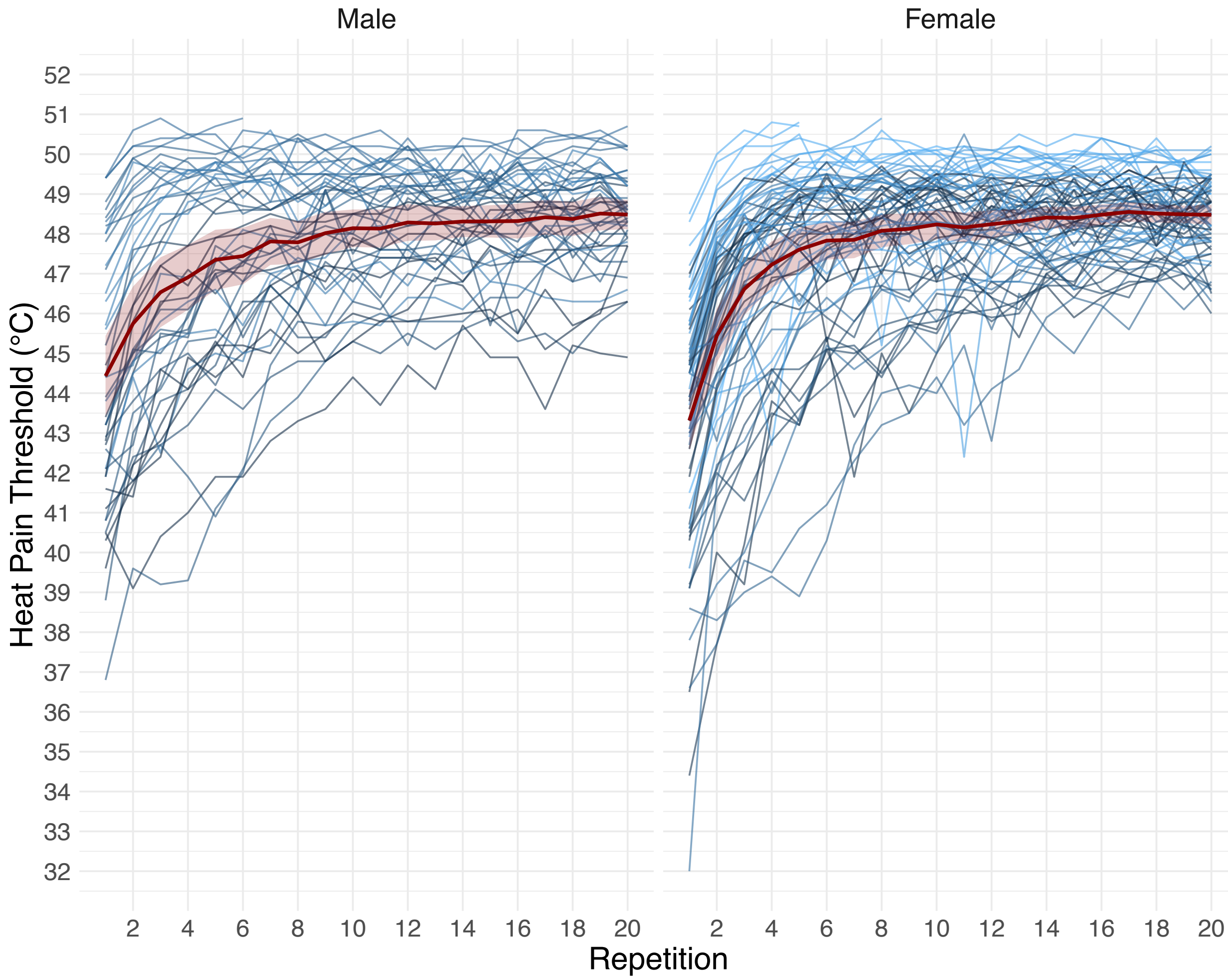

Table S1. Summary of fixed effects from the final asymptotic non-linear mixed-effects model

Parameter component	Predictor (reference)	Estimate, b	Standard Error	t-value (df)	p-value
L	Intercept	48.38	0.20	238.66 (2233)	<0.001***
L	Session 2 (1)	0.25	0.06	4.04 (2233)	<0.001***
L	Session 3 (1)	0.43	0.08	5.57 (2233)	<0.001***
I	Intercept	42.87	0.55	77.78 (2233)	<0.001***
I	Hours since first session	-0.04	0.00	-8.98 (2233)	<0.001***
I	Session 2 (1)	3.16	0.27	11.64 (2233)	<0.001***
I	Session 3 (1)	4.78	0.39	12.15 (2233)	<0.001***
k	Intercept	0.42	0.06	7.15 (2233)	<0.001***
k	Session 2 (1)	0.08	0.01	5.73 (2233)	<0.001***
k	Session 3 (1)	0.17	0.03	5.98 (2233)	<0.001***
k	Side (Right vs Left)	0.04	0.01	6.10 (2233)	<0.001***

****p*<0.001
***p*<0.01
**p*<0.05

Table S2. Differences found from averaging HPT repetitions

Repetitions per group	Session	Side	n	Mean 1	Mean 2	Mean difference	t statistic	df	*P* value	Cohen's *d*
1	1	Left	23	42.52	44.32	1.8	-5.45	22	1.76E-05	1.14
1	1	Right	23	43.1	45	1.89	-8.82	22	1.12E-08	1.84
1	2	Left	22	43.97	45.67	1.7	-10.6	21	6.97E-10	2.26
1	2	Right	22	43.73	45.97	2.24	-5.34	21	2.69E-05	1.14
1	3	Left	14	45.25	46.21	1.47	-6.16	12	4.91E-05	1.71
1	3	Right	14	45.01	47.11	2.1	-8.09	13	1.97E-06	2.16
2	1	Left	23	43.42	45.57	2.15	-7.16	22	3.56E-07	1.49
2	1	Right	23	44.05	46.3	2.25	-10.28	22	7.34E-10	2.14
2	2	Left	22	44.82	47.07	2.25	-15.05	21	1.01E-12	3.21
2	2	Right	22	44.85	47.26	2.4	-7.43	21	2.62E-07	1.58
2	3	Left	14	45.93	47.53	2.06	-8.89	12	1.25E-06	2.47
2	3	Right	14	46.06	48.18	2.12	-8.2	13	1.70E-06	2.19
3	1	Left	23	44.04	46.45	2.41	-7.18	22	3.41E-07	1.5
3	1	Right	23	44.72	46.92	2.2	-7.52	22	1.64E-07	1.57
3	2	Left	22	45.44	47.72	2.27	-12.86	21	2.02E-11	2.74
3	2	Right	22	45.58	47.67	2.08	-8.42	21	3.57E-08	1.8
3	3	Left	14	46.5	48.05	1.97	-7.47	12	7.51E-06	2.07
3	3	Right	14	46.71	48.65	1.94	-7.24	13	6.52E-06	1.94
4	1	Left	23	44.49	46.98	2.48	-8.58	22	1.81E-08	1.79
4	1	Right	23	45.18	47.33	2.16	-7.79	22	9.08E-08	1.63
4	2	Left	22	45.95	48	2.05	-10.35	21	1.06E-09	2.21
4	2	Right	22	46.05	47.94	1.89	-8.02	21	7.89E-08	1.71
4	3	Left	14	46.89	48.38	1.88	-7.09	12	1.26E-05	1.97
4	3	Right	14	47.12	48.91	1.79	-7.36	13	5.53E-06	1.97
5	1	Left	23	44.93	47.32	2.39	-8.82	22	1.13E-08	1.84
5	1	Right	23	45.53	47.58	2.05	-6.64	22	1.12E-06	1.38
5	2	Left	22	46.27	48.2	1.93	-9.89	21	2.35E-09	2.11
5	2	Right	22	46.39	48.06	1.82	-6.76	20	1.41E-06	1.48
5	3	Left	14	47.2	48.57	1.73	-6.12	12	5.20E-05	1.7
5	3	Right	14	47.45	48.82	1.71	-6.45	11	4.74E-05	1.86
6	1	Left	23	45.24	47.55	2.3	-9.15	22	5.96E-09	1.91
6	1	Right	23	45.82	47.74	1.92	-6.15	22	3.44E-06	1.28
6	2	Left	22	46.58	48.32	1.73	-8.64	21	2.36E-08	1.84
6	2	Right	22	46.62	48.21	1.73	-6.37	20	3.22E-06	1.39
6	3	Left	14	47.41	48.62	1.56	-4.83	12	4.09E-04	1.34
6	3	Right	14	47.67	48.7	1.6	-5.64	10	2.17E-04	1.7

7	1	Left	23	45.51	47.7	2.19	-8.86	22	1.05E-08	1.85
7	1	Right	23	46.05	47.91	1.86	-5.67	22	1.06E-05	1.18
7	2	Left	22	46.81	48.39	1.57	-7.68	21	1.56E-07	1.64
7	2	Right	22	46.81	48.33	1.64	-6.05	20	6.49E-06	1.32
7	3	Left	14	47.61	48.69	1.41	-4.49	12	7.44E-04	1.24
7	3	Right	14	47.83	48.74	1.43	-4.87	10	6.47E-04	1.47
8	1	Left	23	45.73	47.82	2.09	-7.97	22	6.29E-08	1.66
8	1	Right	23	46.26	47.86	1.76	-5.38	21	2.43E-05	1.15
8	2	Left	22	46.97	48.43	1.46	-7.21	21	4.18E-07	1.54
8	2	Right	22	46.98	48.45	1.59	-5.58	20	1.85E-05	1.22
8	3	Left	14	47.76	48.73	1.29	-4.24	12	0.00114314	1.18
8	3	Right	14	47.96	48.79	1.31	-4.23	10	0.00175641	1.27
9	1	Left	23	45.95	47.9	1.95	-7.9	22	7.32E-08	1.65
9	1	Right	23	46.4	47.97	1.71	-5.29	21	3.05E-05	1.13
9	2	Left	22	47.11	48.49	1.38	-6.82	21	9.58E-07	1.45
9	2	Right	22	47.13	48.52	1.5	-5.41	20	2.72E-05	1.18
9	3	Left	14	47.89	48.78	1.2	-3.89	12	0.00214775	1.08
9	3	Right	14	48.07	48.8	1.18	-3.91	10	0.0029	1.18
10	1	Left	23	46.12	47.96	1.84	-7.59	22	1.40E-07	1.58
10	1	Right	23	46.55	48.04	1.64	-5.11	21	4.60E-05	1.09
10	2	Left	22	47.24	48.51	1.27	-6.34	21	2.75E-06	1.35
10	2	Right	22	47.26	48.55	1.4	-5.18	20	4.60E-05	1.13
10	3	Left	14	48	48.82	1.11	-3.77	12	0.00269203	1.04
10	3	Right	14	48.16	48.82	1.09	-3.89	10	0.0030052	1.17

Script for participant introduction and familiarisation

Preamble

As you probably know, we are going to measure the temperature at which you first feel pain. Don't worry – these measurements have been used thousands of times by lots of researchers and are very safe.

Remember, if you really do not want to continue with the study, you are free to stop at any time. Please let me know if this is the case.

Equipment

This is the device that we use.

[show Medoc device]

It is very safe.

[Show thermode]

Here is the part that will gradually heat up, which is called a thermode. During the test, this will be held against the skin on the front of each forearm, one after the other.

The thermode will remain at roughly body temperature until the test begins – this is known as the baseline temperature. It will then return back to this baseline temperature after every test.

[Show response button]

Here is the response button that you will hold in your other hand. *At the moment that the heat becomes painful,* you will push the button, and the temperature will then immediately return to back to baseline.

We will be taking 20 measurements on each forearm, with 30 seconds between each measurement. You will be sitting on this chair during the testing. I will put these pillows on your lap so that you are comfortable and can relax your arms.

Familiarisation

So that you get a chance to test out the equipment, we will do a practice test on your ___ leg.

[attach probe to anterior shin and give participant response button]

I will ask you to close your eyes each time the thermode starts to warm up so that you can concentrate on the exact moment that the heat becomes painful.

Remember, *as soon as the heat becomes painful*, you need to push the response button, and the temperature will immediately begin to return back to the baseline temperature.

Script for participant instructions during all HPT sessions

HPT Test- Forearm 1

In a moment, I will begin the testing on your ___ forearm. For the duration of this testing, please try to stay as still as possible.

As before, once the thermode begins to warm up, I will ask you to close your eyes so that you can concentrate on pushing the button *at the moment the heat becomes painful.*

You are welcome to open your eyes during the 30 second break between each test but please keep your eyes facing forwards.

[immediately before hitting start]

Please close your eyes as I will start the test now.

Post-Forearm 1 Test

That concludes the testing on this forearm. We will wait approximately 5 minutes before repeating the same process on your other forearm. During this time, I will move the equipment to this forearm.

HPT Test- Forearm 2

As previous, in a moment, I will begin the testing on your ___ forearm. For the duration of this testing, please try to stay as still as possible.

As before, once the thermode begins to warm up, I will ask you to close your eyes so that you can concentrate on pushing the button *at the moment the heat becomes painful.*

You are welcome to open your eyes during the 30 second break between each test but, again, please keep your eyes facing forwards.

[immediately before hitting start]

Please close your eyes as I will start the test now.
